# Bullying experiences and mothers’ responses to bullying of children with autism spectrum disorder

**DOI:** 10.1007/s44202-022-00045-3

**Published:** 2022-08-15

**Authors:** Budor H. Saigh, Nizar H. Bagadood

**Affiliations:** Department of Special Education, College of Education, Umm Al-Qura University, Makkah, Saudi Arabia

**Keywords:** Autism spectrum disorder, Bullying, Parental support

## Abstract

Despite the fact that children with disabilities generally have been shown to be at a greater risk of bullying, which include physical attacks and threats, being picked on and teased and verbal abuse, there is still a need to understand bullying of children with autism spectrum disorder (ASD) and the strategies used by parents to support them in tackling this issue. This study aims to investigate the type and level of occurrence of bullying and parental support for children aged five with ASD who suffer from bullying. This study used both quantitative and qualitative analysis; a questionnaire, distributed to mothers of children with ASD, adopted from the Bullying for ASD Survey developed by Chen and Schwartz (Focus Autism Other Dev Disabil 27(4):200–212, 2012) measured types and frequency of bullying and parental support. An open-ended question was added to the survey to enable parents to write about the ways in which their child was bullied and the ways in which they supported their child. The results showed that while children suffered from all types of bullying, especially being picked on, being excluded and called names. Parental support showed a higher prevalence of involving peers and teachers for the prevention of bullying as well as avoiding strategies which included avoiding bullies and none of the mothers encouraged their children to stand up to bullies.

## Introduction

The characteristics of individuals diagnosed with autism spectrum disorder (ASD) include communication and social difficulties, extreme sensitivity to most sensory stimuli, and repetitive behaviours [1]. In addition, they may have problems with non-verbal communication and taking turns in conversation. The estimated prevalence of ASD in the UK has been stated to be 1–3% of the population [2]. Children with ASD are at an increased risk of bullying at school [3, 4] and bullying is more likely to occur significantly earlier in children with ASD than in children without ASD [5]. In fact, a significant relationship between the severity of ASD and the risk of being bullied has been found [6]. Bullying is defined as behaviours carried out and repeated with the intention of causing emotional distress or physical harm [7]. Various factors contribute to bullying of children with ASD, for example, different levels of social understanding [8] and stereotypes and negative beliefs held by neurotypical children resulting in discrimination and prejudice [9]. Hsiao et al. [10] found that psychopathologies mediated the link between ASD and bullying in addition to the direct effect of ASD on bullying victimisation.

Research findings have indicated that bullying can have a variety of detrimental outcomes for the child victim with ASD, such as problems with mental health, suicidal thoughts, and damaged self-esteem [11–13]. It was found that in groups of children in inclusive schooling, 75% were bullied, of which 40% felt rejected as a result of their victimisation [14]. School refusal is another problem for children with ASD, Ochi et al. [5] found that school refusal occurred earlier in children with ASD than in those children without. Children in inclusive school settings have been found to be more likely to be bullied than children in special education schools [15–17].

The study makes a contribution to understanding the type of bullying and frequency of bullying experienced by children and how mothers cope, as current research is often centred around much older children and adolescents and the coping strategies of mothers has been little studied. Towards the development of strategies to support children there has been much work to understand bullying, which has included consideration of the experiences of parents. The objective of the study is to reveal how mothers support children at this age who are victims of bullying. This study considers that a beneficial contribution to such knowledge would be to reveal the approaches that parents use to support their children with ASD who are victims of bullying. The study reveals these approaches which can contribute to the development of future strategy for younger children with ASD.

## Literature review

Hwang et al. [4] describe the issue of bullying of children with ASD as significant, highlighting their greater risk of bullying exposition compared to other children in the community. They propose that there is a need to effectively recognise children with ASD and the danger of school bullying and develop strategies and interventions to support them. Chou et al. [18] who examined the role of bullying involvement in depression and anxiety among adolescents, draw analogous conclusions when commenting on such issues among individuals with ASD who have experienced bullying, pointing out that this topic should be regularly examined.

Parental involvement in children with ASD who are victims of bullying has been addressed from a number of different perspectives. Mademtzi et al. [19] examined the parental experiences of caring for female children with ASD and found that parents said they suffered the same issues as males including difficulty in social interactions. The general experience of parents caring for children with ASD has included bullying as a challenged faced by parents [20]. Views from parents have also been sought to identify the types of bullying that their children. Kloosterman et al. [21] examine the parental agreement with children’s experience of bullying where it was found that there was a high level of agreement between parents and children with ASD than other children. This suggests that parents of children with ASD are more aware of the types bullying that their children are victims of. Ashburner et al. [22] examined the perception of parents of how bullying affected their children, parental concerns included the effects of bullying on self-esteem, attendance, social participation, mental health, behaviour and academic performance.

Much of literature about children with ASD and bullying focus on strategies of support generally including the prevention of bullying [23]. Researchers have contributed through identifying risk factors that are associated with vulnerability and protection [3]. Interventions from educational establishments and from parents’ recommendations are provided based on the findings where parents perceptions are sought. Where parents are consulted it is for their perspectives on bullying of their children with ASD [15]. VanOrmer et al. [24] found that parental tolerance of children’s behaviour with ASD was important in shaping the discipline strategy that parents used, their study found that parents of children with ASD were more tolerant of their child’s disruptive behaviour. Importantly, Kloosterman et al. [21] bring attention to the fact that parents are often involved in helping their children, however, this is limited to asking from parents the type and frequency of their child’s bullying. This supports the position of the present study that there is a need to understand more than just types of bullying that are experienced from the parents’ perspective, there is a need to understand further the strategies that parents use. Yoo et al. [25] examine the use of a parent-assisted social skills training program for teens with ASD which was shown to be effective in improving social skills of adolescents in North America. Schiltz et al. [26] examined the link between the challenging behaviour of youth with ASD and parental stress and involvement and found that stress has a negative impact on parental involvement, motivating the need to consider the whole family system in research on young people with ASD.

A far-reaching study by Carrington et al. [27] investigated the perspectives of students with ASD and their parents in terms of dealing with bullying in an Australian school and concluded with a range of recommendations and strategies, such as raising awareness of bullying; developing rules that can be more clearly linked to school, students, and their parents; improving communication and relationships within families to overcome bullying; and providing coping strategies to deal with bullying. This study was more comprehensive in that it included both children and parents to derive communication and coping strategies [27], however the children were between 11 and 16 years of age, the weakness being that the wide age cohort would include children and adolescents whereby experiences and coping strategies could differ.

Laugeson et al. [28]  asked a sample of 33 teenagers with ASD to complete, with parental help, assignments designed to teach a range of social skills. The researchers found that the intervention increased the quality of the teenagers’ friendships and their social etiquette. Unfortunately, the researchers did not comment on the intervention’s effects on bullying. Additionally, the intervention did not teach the teenagers how to respond to bullying.

These studies are focussed on teenagers and students and such recommendations for interventions may not be appropriate for younger children. Therefore, there is a need to reveal how young children are supported. It is noticeable in the literature that studies about parents’ involvement or experiences are often related to older children and adolescents, high school pupils and adolescents [25, 26, 29] students [22, 27] older children between the ages of 9 and 14. There have been few studies that have considered much younger children, however, these studies also included adolescents and adults [19].

Furthermore, few studies have been conducted to understand the parental support of young children with ASD who are victims of bullying. Zeedyk et al. [30] commented on the dearth of research in this area, noting that strategies are required not only to support the social communication skills of children with ASD, but also to empower them to sustain healthy peer relationships and avoid being at risk of victimisation. Furthermore, while there has been research on parenting behaviour among parents who have children with ASD, the degree of involvement or engagement has not received much attention (Schiltz et al. 2018).

To the best of this researcher’s knowledge, no studies have reported on the topic of parental support solely in aiding children in the 5 years age cohort with ASD as a response to bullying. The present study thus aims to investigate how parents support their children with ASD, at age 5 in primary school, to respond to bullying. Parents are asked about frequency of bullying, types of bullying in addition to the support parents provide for their children to help them respond to bullying.

## Method

### Participants

Seven mothers agreed to take part in the study. Their children were 5 years old, had been diagnosed with ASD (Mothers were asked if their child had been diagnosed with ASD, with the following question: ‘Has a doctor or health professional ever told you that [your child] has autism … or autistic spectrum disorder?’ ([31], p. 2310)) and were attending a reception class in an inclusive primary school. Three of the children were male and four were female. They were given pseudonyms as follows: Anne, Billie, Cathy, Daphne, Elizabeth, Thomas, and John.

### Instruments

The aims of the study were to reveal the experiences of bullying from the perspective of mothers including type and frequency of bullying and how mothers support their child where their children are being bullied. For the type and frequency of bullying the Bullying for ASD Survey developed by Chen and Schwartz [32] was adopted in this study for the mothers to complete. The survey is suitable for measuring bullying for children with ASD from the parental perspective and has been previously validated by Chen and Schwartz [32]. This survey was informed by a number of different surveys including Olweus Bully/Victim Questionnaire by Olweus [33] and the Self-Report Victimisation Scale by Ladd and Kochenderfer-Ladd [34]. Importantly, the survey includes a parent version in addition to teacher and student version [32]. The parent version of the questionnaire asked questions related to the same constructs that children are asked. In the original survey there were ten types of bullying included the same items were included in the present study.

A four-point Likert scale of frequency was used in the questionnaire. For the first part of the questionnaire parents were asked about the type and frequency of bullying that their children experienced (Table 1). An open-ended question was added to the end of this section to allow the mothers to write about any other ways in which their child was being bullied at school. For the second part of the questionnaire mothers were questioned about the type and frequency of support that they gave children (Table 2). Mothers were also given the opportunity to write about other ways that they support their children.

**Table 1:** Type and occurrence of bullying at school

Has a doctor or health professional ever told you that [your child] has autism … or autistic spectrum disorder?	Yes 7	No 0		
My child has experienced the following:	Never	Once or twice	Three or four times	Five or more times
Picked on	0	0	2	5
Excluded	0	2	1	4
Laughed at	1	3	1	2
Kicked or hit	1	4	2	0
Threatened	0	3	4	0
Teased	0	0	6	1
Negative comments	0	1	4	2
Destroyed possessions	5	2	0	0
Called names	0	0	4	3
Spread rumours	0	0	2	5

**Table 2:** Parents’ support their children about bullying at school

I speak to my child about the following:	Never	Once or twice	Three or four times	Five or more times
Talk with your child about inappropriate and appropriate behaviour from other children at school	0	4	1	2
Ask your child to stay close to teachers during play times	0	3	3	1
Ask child not to take any valuables with them to school	1	0	2	4
Ask your child to walk with a friend or a teacher when they walk to different rooms at school	0	2	5	0
Discuss with your child their concerns about being with their peers at school	0	4	2	1
Discuss how your child can get help by approaching teachers	0	2	2	3

### Procedure

The researcher contacted an associate who had a 5-year-old child with ASD who had experienced bullying in primary school. Six additional mothers were identified by of word of mouth from the initial contact; this was snowball sampling where the participants were identified from the personal contacts and social network of the earlier participants [35]. All participants had a 5-year-old child with ASD, who had experienced bullying at school. A consent form was sent, signed, and returned (see Appendix A) via email, by each participant. The two survey questionnaires were emailed to the participants, completed, and emailed back to the researcher.

## Results

### Parents’ reports of types of bullying at school using the bullying for ASD Survey

Table 1 displays the different types of bullying at school, according to mothers’ responses to the Bullying for ASD Survey questionnaire. A total of five children had been picked on by their peers on more than five occasions. Four children had been excluded by their peers, also on more than five occasions. Five children had also had rumours spread about them at least five times. Seven had been called names; for four children this had happened three or four times, and for three children it had happened five or more times. One had been teased on five or more occasions. A total of five children had been kicked or hit, three once or twice and two on three or four occasions. Only two out of the seven children had had their property destroyed, on one or two occasions, whereas six children had been teased on three or four occasions, and one had been teased five or more times.

### Parents’ reports of other types of bullying at school

In answer to the open-ended question included in the Bullying for ASD Survey, ‘Please could you state any other ways in which your child has been bullied with an example’, six mothers responded with the following examples of bullying experienced by their child:

*Other children bully Thomas by frightening him. They say things like, ‘I don’t think your mum is going to pick you up today’. (*Thomas’ mother)

*During PE lessons they hide her coat, shoes, laces, socks. They have even hidden her lunchbox.* (Elizabeth’s mother)

*They tell lies about John. They say something like, ‘John swore at me’. John ends up being told off for something he hasn’t done. The head teacher said he has got to stand up for himself.* (John’s mother)

*They are always tricking her into a difficult situation. The bully, and there are usually two of them, said once, ‘Kalid has lost his chocolate bar, oh look, Cathy has got it in her bag’.* (Cathy’s mother)

*Other children want the best seat at the back of the class, and if Anne sits on that seat she is told to move from that seat.* (Anne’s mother)

*Billy often talks too much when others have had enough of the conversation. Then they tease him and call him stupid.* (Billy’s mother)

### Parents’ reports of the ways they support their child to respond to bullying, using the Parents’ Support Survey Questionnaire

The mothers in the sample agreed that they provided their children with ways to respond to bullying, displayed in Table 2. A total of four mothers agreed that they asked their child five or more times ‘not to carry any valuables with them to school’. Five asked their children three or four times ‘to walk with a friend when they walk to different rooms at school’. Four reported that they discussed once or twice and two had talked to their child on five or more occasions ‘about inappropriate and appropriate behaviour from other children at school’. Four mothers had discussed how their child could ‘get help by approaching teachers’.

### Parents’ reports of other ways in which they support their child to respond to bullying

In answer to the open-ended question included in the Parents’ Support Survey questionnaire, ‘Please could you state the ways in which you support your child to respond to bullying when they are at school’, four mothers responded to the question. The idea of using teachers for support was suggested in terms of being near the teacher to avoid bullying.

*I try to remain calm when I’m talking to Daphne about bullying. I have talked to her about approaching teachers, not to talk to them, just to stand near them. That seems to work for her. The bully keeps away from her. If I show that I am worried she picks up on that.* (Daphne’s mother)

*I have contacted the school several times to ask them to punish bullies. I can only tell John to go to the teacher when other children make him feel uncomfortable or are being abusive towards him, otherwise they still bully him.* (John’s mother)

To get the child to concentrate on their schoolwork was another way of parental support for the child:

*Anne doesn’t want to go to school sometimes. I’ve had to boost her self-confidence and remind her that she can concentrate on subjects because she has ASD, and she could do that better than children without ASD. If she was feeling low, I don’t think she would have been able to cope with the pressure of bullying.* (Anne’s mother)

Avoidance was a strategy that was revealed in this study, that parents have suggested the child simply ignores confrontation to avoid bullying:

*Billie switches off when he feels overwhelmed with others. He prefers to be on his own. I have suggested it is okay for him to do this because he avoids confrontation.* (Billie’s mother)

## Discussion

The study set out to investigate the type and frequency of bullying of children with ASD from the perspective of mothers and how mothers supported their children who are victims of bullying. This was achieved with a questionnaire where the quantitative element measured types and frequency of bullying and types of parental support and a qualitative open question section that allowed mothers to elaborate on how they support their children. In their responses to the bullying survey, all mothers reported that their children had been bullied on at least three or four occasions, and many had been bullied on five or more occasions. The children had experienced all ten types of bullying set out in the bullying survey. A large majority of the children had been picked on, had rumours spread about them, and had been excluded. The mothers reported additional types of bullying experienced by their child, including being frightened, having their possessions stolen, others telling lies about them, and being tricked into difficult situations.

All the mothers reported that they provided support in agreement with the six items on the support survey. They also reported additional ways that they supported their child to help them respond to bullying which included being calm about the situation, Daphne’s mother reported that her support involved being calm and that this was important when she was advising her daughter on how to respond to bullying behaviour. Parental stress can act as a moderator between anxiety and bullying victimisation. In a study of 101 mothers of young adults and teenagers with ASD, it was found that if parents were highly stressed, children with ASD experienced more anxiety related to bullying victimisation [36]. Daphne’s mother was able to support her daughter to respond to bullying by suggesting she position herself within close proximity to teachers for protection, this was to boost self-confidence.

Anne’s mother reported that her daughter did not always want to go to school. Research has noted that school avoidance is common among children with ASD when they are victims of bullying [5, 37]. Anne’s mother also reported that her daughter had low self-esteem because of the bullying; consequently, she provided support to increase Anne’s sense of self-esteem to help her feel good about going to school [38].

Billie’s mother reported that he switches off when he feels overwhelmed at school; when he does this, he avoids confrontation. It seems that Billie uses an avoidant strategy when he feels like he is not in control of a situation. Dated but relevant research has noted that this is common among children with ASD who encounter frequent bullying at school [39].

John’s mother was frustrated with the lack of punishment being given out to bullies and had appealed to her son’s school to punish bullies. This is a common parent request. Past research has reported that other parents of children with ASD have contacted their child’s school with this request [27]. The researchers recommended that school practices and policy should set out ways in which they can reduce the incidence of bullying in inclusive and special education.

None of the parents suggested to their child that they stand up to the bully. Rather, the parents’ support strategies were associated with avoidant strategies, particularly approaching a teacher, and telling them about the situation or being in close proximity to a teacher when moving about the school. These support strategies may be considered as suggestions of passive behaviours, which may not be effective against bullying. When children engage in particular strategies to cope with bullying, such as helpless behaviour, fighting back, or passive behaviours, that these strategies are the least effective in coping with bullying [40]. It may be that the mothers do not wish their children to get too involved with the bully, because of the possible repercussions. Zablotsky et al. [15] found that, out of a total of 1221 parents of children with ASD who completed their survey, found that only 18.5% reported that their children had an emotional outburst or were provoked and fought back.

Where this study has contributed to the current research is that it has investigated how mothers support their children with ASD when they are bullied. This approach takes ideas from parents rather than offering strategies to parents, or just simply investigating frequencies of bullying from parents. The contribution here is that strategies are taken from parents who are perhaps in a better position, through experience, to offer recommendations, something that has been missing in the research. Furthermore, there has been a lack of research for younger children alone, this study has provided insight into an age group where experience of bullying and parental support may be different than for older children and adolescents.

## Conclusion and recommendations

This investigation aimed to assess how parents support their child with ASD, at age five, in primary school, to respond to bullying. Children with ASD are a vulnerable group at greater risk of bullying victimisation. Since parents have a responsibility to protect their children with ASD, continued efforts are needed via research to make practical recommendations by identifying coping strategies associated with bullying and parenthood. An important task for future research is to examine and recommend coping strategies for children with ASD. Specifically, the development of future intervention should consider the experienced positions and approaches of the parents dealing with bullying of children with ASD. In light of this it is recommended that there is further investigation into the effectiveness of these parental approaches.

The generalisability of these results is subject to certain limitations. For instance, observation of real-life bullying in a school context or children’s responses to bullying in that setting was not the focus of this research, since the researcher wanted to avoid causing distress or harm to the children who were discussed in this study. Thus, the research data derived solely from mothers’ responses to the survey questionnaire and their comments in response to the two open-ended questions provided at the end of the survey questionnaires. The research was carried out with questionnaires sent and returned by email, to adhere to the requirements of the government’s rules on social distancing and staying at home during the Covid-19 pandemic. More detailed data might have been obtained if the researcher had been able to interview the mothers in person. Additionally, the mothers’ reports of their child’s experience of bullying and the support they provided, could not be further verified by the researcher. Due to practical considerations the research sample was relatively small, a future study should include a larger sample.

Finally, it was a contribution of the study that a specific age was considered, a future study could investigate if there are differences in experiences and support by parents between different age groups. This would contribute to more age-appropriate strategies to support these children.

## Appendix A: Parents’ Consent Form

### Project Title: Mothers’ responses to bullying of their 5-year-old children with autism spectrum disorder attending a reception class in an inclusive primary school

**Dear participant,**

This questionnaire is investigating ways in which parents support their child with ASD to respond to bullying.

This research is being carried out by [                                ] under the supervision of [                                    ].

You will not be asked to provide your name, but you may be asked to provide some demographic information for analysis purposes. Data collected through this questionnaire will be aggregated and you will not be individually identifiable in any reports or publications from this research. All information collected will be kept securely and will only be accessible by [           ].

For more information about your rights as a participant in this research please download a copy of the participant information sheet which can be found at this link [                ].

We would be very grateful for your participation in this study. If you need to contact us in future, please contact me (              ) or [                ] (                   ).

You can also contact us in writing at:.

Yours,

[                  ]

### Statement of Consent

By submitting a completed version of this questionnaire, you are consenting to the following:

- I agree to participate in the research project, ‘Parental support for young children (aged 5) with autism spectrum disorder, to respond to bullying’ being carried out by [                     ]
- This agreement has been given voluntarily and without coercion.
- I have been given full information about the study and contact details of the researcher(s).
- I have read and understood the information provided above.
- I have had the opportunity to ask questions about the research and my participation in it.

## Appendix B: Parents’ bullying survey for ASD

**Table:**

‘Has a doctor or health professional ever told you that [your child] had Autism … or autistic spectrum disorder?	Yes	No		
My child has experienced the following:	Never	Once or twice	Three or four times	Five or more times
Peers have picked on them				
Peers have excluded them from activities				
Peers have laughed at them				
Peers have kicked or hit them				
Peers have threatened them with physical harm				
Being made fun of or teased by peers				
Peers have made fun of them				
Peers have made negative comments about them				
Peers have destroyed their personal				
Peers have called them names				
Peers have spread rumours about them				
Peers have picked on them				
Please could you state any other ways in which your child has been bullied (and an example)				

## Appendix C: Parents’ Support Survey Questionnaire

**Table:**

I speak to my child about the following:	Never	Once or twice	Three or four times	Five or more times
Talk with your child about inappropriate and appropriate behaviour from other children at school				
Ask your child to stay close to teachers during play times				
Ask child not to carry any valuables with them to school				
Ask your child to walk with a friend or a teacher when they walk to different rooms at school				
Discuss with your child their concerns about being with their peers at school				
Discuss how your child can get help by approaching teachers				
Please could you state the ways in which you support your child to respond to bullying when they are at school				

Thank you for your participation in this study.

You are reminded that by submitting a completed version of this questionnaire you are agreeing to participate in this research.

If you have any queries, please contact me (                  ) or [                  ].

(                ).

You can also contact us in writing at:

Yours,

[                  ].
